# Severe Methanol Intoxication: A Case Report on Clinical Management and Recovery

**DOI:** 10.51894/001c.144453

**Published:** 2025-09-30

**Authors:** Zairah Fatima

**Affiliations:** 1 Department of Internal Medicine Detroit Medical Center-Sinai Grace Hospital

## 77

Methanol intoxication is a life-threatening condition characterized by severe anion gap metabolic acidosis, CNS toxicity, specifically bilateral putaminal hemorrhage, and potential optic neuropathy if not promptly managed. It often results from accidental or intentional ingestion of methanol-containing products, such as windshield wiper fluid.

A 50-year-old male presented to SGH emergency department with altered mental status after being picked up by EMS. The patient initially reported generalized body pain and endorsed ingestion of windshield washer fluid 36 hours ago. Shortly after, his mentation deteriorated further, exhibiting tachypnea, tachycardia, bilateral nonreactive pupils, absent vision and foaming at the mouth. Initial labs revealed severe anion gap metabolic acidosis with a significantly low bicarbonate, significant hyperkalemia, and a toxic methanol concentration of 392 mg/dL. Initial CT of the head showed no acute abnormalities, although repeat CT on day 3 revealed large areas of low attenuation involving the putamen, external capsule, and subcortical white matter. In the ED, the patient received intubation, bicarbonate, Ativan, and Toxicology started him on fomepizole, folinic acid, and N-acetylcysteine and urgent hemodialysis was initiated, reducing methanol levels from 392 to 20 mg/dL and correcting metabolic abnormalities. MRI confirmed necrosis of bilateral lateral putamina/external capsules and subcortical white matter. Ophthalmology evaluation indicated toxic optic neuropathy with blurred optic disc margins. Rehabilitation included physical therapy and occupational therapy were consulted for persistent visual and cognitive deficits. Within one week of admission, he was alert, oriented, and cooperative, with improved motor coordination and vision. He was transferred to the rehabilitation unit for two weeks and discharged home in stable condition under supervision with outpatient ophthalmology and neuropsychiatry follow-up for visual problems, language deficits, cognitive issues, and problem-solving challenges.

Despite the delayed presentation of the patient more than 24 hours post-methanol ingestion and the need for mechanical ventilation, prompt diagnosis and the initiation of aggressive, targeted treatment significantly facilitated quick recovery and markedly improved the likelihood of survival. Multidisciplinary care, including hemodialysis and targeted antidotes, can also significantly improve survival and functional outcomes. Long-term rehabilitation care is also crucial for residual neurological and cognitive impairments.

